# Glutathione S-transferase M1 null genotype meta-analysis on gastric cancer risk

**DOI:** 10.1186/1746-1596-9-122

**Published:** 2014-06-19

**Authors:** Xianhong Meng, Yong Liu, Bona Liu

**Affiliations:** 1 Department of Gastroenterology, Affiliated to the Fourth Hospital of Harbin Medical University, Harbin 150001, China; 2 Department of Oncology, People’s Liberation Army No.202 Hospital, Shenyang 110000, China

**Keywords:** GSTM1, Polymorphism, Gastric cancer, Risk, Meta-analysis

## Abstract

**Background:**

Glutathione S-transferases (GSTs) have proved to be involved in the detoxifying several carcinogens and may play an important role in carcinogenesis of cancer. Previous studies on the association between Glutathione S-transferase M1 (GSTM1) polymorphism and gastric cancer (GC) risk reported inconclusive results. To get a precise result, we conducted this present meta-analysis through pooling all eligible studies.

**Methods:**

A comprehensive databases of Pubmed, Embase, Web of Science, and the Chinese Biomedical Database (CBM) were searched for case–control studies investigating the association between GSTM1 null genotype and GC risk. Odds ratios (OR) and 95% confidence intervals (95% CI) were used to assess this possible association. A χ2-based Q-test was used to examine the heterogeneity assumption. Begg’s and Egger’s test were used to examine the potential publication bias. The leave-one-out sensitivity analysis was conducted to determine whether our assumptions or decisions have a major effect on the results of present work. Statistical analyses were performed with the software program STATA 12.0.

**Results:**

A total of 47 eligible case–control studies were identified, including 6,678 cases and 12,912 controls. Our analyses suggested that GSTM1 null genotype was significantly associated with increased risk of GC (OR = 1.186, 95% CI = 1.057-1.329, P_heterogenetiy_ = 0.000, P = 0.004). Significant association was also found in Asians (OR = 1.269, 95% CI = 1.106-1.455, P_heterogenetiy_ = 0.002, P = 0.001). However, GSTM1 null genotype was not contributed to GC risk in Caucasians (OR = 1.115, 95% CI = 0.937-1.326, P_heterogenetiy_ = 0.000, P = 0.222). In the subgroup analysis stratified by sources of controls, significant association was detected in hospital-based studies (OR = 1.355, 95% CI = 1.179-1.557, P_heterogenetiy_ = 0.001, P = 0.000), while there was no significant association detected in population-based studies (OR = 1.017, 95% CI = 0.862-1.200, P_heterogenetiy_ = 0.000, P = 0.840).

**Conclusion:**

This meta-analysis showed the evidence that GSTM1 null genotype contributed to the development of GC.

**Virtual Slides:**

The virtual slide(s) for this article can be found here: http://www.diagnosticpathology.diagnomx.eu/vs/1644180505119533.

## Background

Multiple lines of evidence suggested both cumulative effect of environmental risk factors and genetic susceptibility of the individual contributed to the development of the cancers [1]. The gene-environment interaction in carcinogenesis is also well reflected by metabolic enzymes involved in the inactivation and/or detoxification of environmental carcinogens. Most of the carcinogens are metabolically inactivated by detoxification enzymes. Therefore, inherited variations in genes encoding the carcinogen-metabolizing enzymes may alter enzymatic activity and subsequently the carcinogens activation and/or deactivation [2]. Individual susceptibility to cancer is likely to be affected by the genotypes of biotransformation enzymes which represent significant ethnic differences in the frequency of alleles [3].

Human glutathione S-transferases (GSTs) are phase II metabolizing enzymes that play a key role in protecting against cancer by detoxifying numerous potentially cytotoxic/genotoxic compounds [4]. The genes encoding the three major GST isoenzymes, GSTM (mu) 1, GSTT (theta) 1, and GSTP (pi) 1, widely expressed along the human gastrointestinal tract [5], are highly polymorphic. Among the GST isoforms, glutathione S-transferase M1 (GSTM1) is of particular interest and important because it possesses a present/null polymorphism and the null genotype has a complete absence of GSTM1 enzyme activity. It has been observed that GSTM1 null may affect individual susceptibility to cancer [6]. Up to now, numerous researches about the relationship between the polymorphism of GSTM1 null genotype and GC susceptibility have been conducted. However, the findings are controversial due to different reasons including the populations selected and their ethnicities. A recent meta-analysis of 15 studies suggested no association between the GSTM1 polymorphism and GC susceptibility was found [7]. When they performed the meta-analysis, the pooled sample size was relatively small and not enough information was available for more exhaustive subgroup analysis. Since then, additional several studies with a large sample size about this polymorphism on GC risk have been reported, which would greatly improve the power of the meta-analysis. In order to get a more precise result, we conducted this present meta-analysis.

## Methods

### Search strategy for eligible studies

We conducted a comprehensive search through the Pubmed, Embase, Web of Science, and Chinese Biomedical Data-base (CBM) databases for studies assessing the association between GSTM1 null genotype and GC risk. The literature strategy used the following keywords: (“Glutathione S-transferase M1”, “GSTM1” or “GSTM”) and (“gastric cancer”, “gastric carcinoma”, “stomach cancer” or “stomach carcinoma”). There was no sample size and language limitation. We evaluated all associated publications to retrieve the most eligible literatures. All references cited in the included studies were also hand-searched and reviewed to identify additional published articles not indexed in common databases. Of the studies with overlapping data published by the same authors, only the most recent or complete study was included in this meta-analysis.

### Inclusion and exclusion criteria

The inclusion criteria of eligible studies were as following: (1) Evaluate the GSTM1 polymorphism and GC risk; (2) Only the case–control studies were considered; (3) The paper should clearly describe the diagnoses of GC and the sources of cases and controls; (4) The controls were gastric cancer-free individuals; (5) Reported the frequencies of GSTM1 polymorphism in both cases and controls or the odds ratio (OR) and its 95% confidence interval (95% CI) of the association between GSTM1 null genotype and GC risk. The exclusion criteria were: (1) none case–control studies; (2) control population including malignant tumor patients; and (3) duplicated publications.

### Data extraction

Relevant data were extracted from all the eligible studies independently by two reviewers, and disagreements were settled by discussion and the consensus was reached among all reviewers. The main data extracted from the eligible studies were as following: the first author, year of publication, ethnicity, genotype method, source of the controls, total numbers of cases and controls, the genotype frequency of GSTM1 polymorphism. Different ethnicities were mainly categorized as Caucasians, Asians, Africans, and Mixed. If a study did not specify the ethnicity or if it was not possible to separate participants according to such phenotype, the group was termed “mixed”. For studies including subjects of different ethnic populations, data were collected separately whenever possible and recognized as an independent study.

### Quality assessment

Quality of eligible studies in present meta-analysis was assessed using the Newcastle Ottawa scale (NOS) as recommended by the Cochrane Non-Randomized Studies Methods Working Group. This instrument was developed to assess the quality of non-randomized studies, specifically cohort and case–control studies [8]. This instrument was developed to assess the quality of nonrandomized studies, specifically cohort and case–control studies. Based on the NOS, case–control studies were judged based on three broad perspectives: selection of study groups (1 criterion), comparability of study groups (4 criteria), and ascertainment of outcome of interest (3 criteria). Given the variability in quality of observational studies found on our initial literature search, we considered studies that met 5 or more of the NOS criteria as high quality (http://www.ohri.ca/programs/clinical_epidemiology/oxford.asp) [9].

### Statistical methods

We examined the association between GSTM1 null genotype and GC risk by calculating pooled odds ratio (ORs), 95% confidence intervals (95% CI), and the significance of the pooled OR was determined by the Z-test. To assess the heterogeneity among the included studies more precisely, both the chi-square based Q statistic test (Cochran’s Q statistic) to test for heterogeneity and the I^2^ statistic to quantify the proportion of the total variation due to heterogeneity [10,11]. If obvious heterogeneity existed among those included studies (P < 0.05), the random-effect model (DerSimonian and Laird method) was used to pool the results [12]. When there was no obvious heterogeneity existed among those included studies (P > 0.05), the fixed-effect model (Mantel-Haenszel’s method) was used to pool the results [13]. Moreover, subgroup analyses were performed to test whether the effect size varied by the ethnicity and the source of control population. The kinds of ethnicity were mainly defined as Caucasians, Asians. Publication bias was investigated with the funnel plot and its asymmetry suggested risk of publication bias. To evaluate the published bias, we used Begg’s [14] and Egger’s [15] formal statistical test and by visual inspection of the funnel plot. Furthermore, the leave-one-out sensitivity analysis was conducted to determine whether our assumptions or decisions have a major effect on the results of the review by omitting each study [16]. All statistical tests for this meta-analysis were performed with STATA (version 12.0; Stata Corporation, College Station, TX). A P value less than 0.05 was considered statistically significant, and all the P values were two sided.

## Results

### Study characteristics

**Figure 1:**
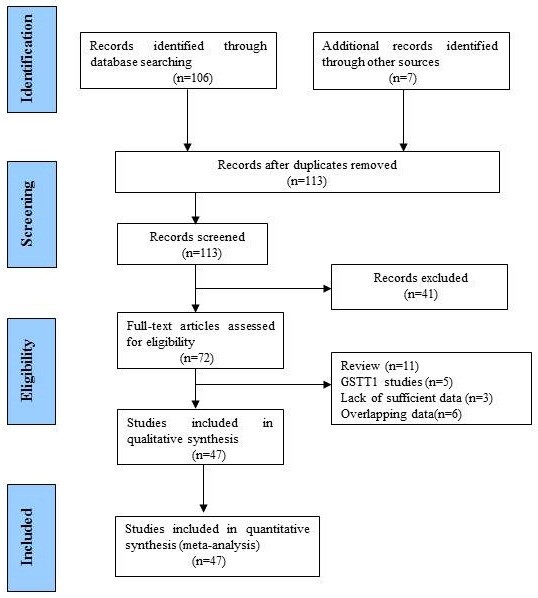
Flow chart of study selection.

There were 113 relevant abstracts identified by searching the key words, and 41 studies were firstly excluded after the careful review of the abstracts, leaving 72 studies for full publication review (Figure 1). Of those 72 studies, 25 studies were excluded (6 for containing overlapping data, 11 for reviews, 3 for without adequate data, and 5 for on GSTT1 polymorphism). Table 1 listed the main characteristics of eligible studies included in this meta-analysis. There are 47 case–control studies, including 6,678 cases and 12,912 controls met the selection criteria [2,17-62]. Among the 47 studies, 24 studies are of Caucasians and 23 studies are of Asians. There are 25 studies of hospital-based controls and the rest are population-based controls.

**Table 1:** Main characteristics of all the eligible studies in this meta-analysis

**First author**	**Year**	**Ethnicity**	**Control source**	**Sample size**		**Case**		**Control**	
				**Case**	**Control**	**Present**	**Null**	**Present**	**Null**
Strange et al.	1991	Caucasian	Hospital-based	19	49	5	14	29	20
Harada et al.	1992	Asian	Population-based	19	84	14	5	44	40
Kato et al.	1996	Asian	Hospital-based	64	120	34	30	59	61
Katoh et al.	1996	Asian	Population-based	139	126	60	79	71	55
Deakin et al.	1996	Caucasian	Hospital-based	136	577	64	72	261	316
Enders et al.	1998	Caucasian	Hospital-based	51	35	23	28	22	13
Martins et al.	1998	Caucasian	Hospital-based	148	84	77	71	40	44
Oda et al.	1999	Asian	Hospital-based	147	112	56	91	57	55
Cai et al.	1999	Asian	Population-based	95	94	35	60	51	43
Setiawan et al.	2000	Asian	Population-based	87	419	45	42	207	212
Lan et al.	2001	Caucasian	Population-based	347	426	180	167	204	222
Saadat et al.	2001	Caucasian	Population-based	42	131	16	26	78	53
Gao et al.	2002	Asian	Population-based	153	223	63	90	90	133
Wu et al.	2002	Asian	Hospital-based	356	278	183	173	142	136
Sgambato et al.	2002	Caucasian	Hospital-based	8	100	3	5	47	53
Choi et al.	2003	Asian	Population-based	80	177	34	46	82	95
Roth et al.	2004	Asian	Population-based	90	454	66	24	309	145
Suzuki et al.	2004	Asian	Hospital-based	145	177	58	87	93	84
Colombo et al.	2004	Mixed	Population-based	100	150	53	47	88	62
Lai et al.	2005	Asian	Hospital-based	123	121	50	73	66	55
Li et al.	2005	Asian	Hospital-based	100	62	33	67	36	26
Mu et al.	2005	Asian	Population-based	196	393	69	127	158	235
Nan et al.	2005	Asian	Hospital-based	400	614	149	251	254	360
Shen et al.	2005	Asian	Hospital-based	142	675	41	71	314	361
Palli et al.	2005	Caucasian	Population-based	175	546	85	90	271	275
Tamer et al.	2005	Caucasian	Hospital-based	70	204	30	40	116	88
Nan et al.	2005	Asian	Hospital-based	107	220	34	73	90	130
Hong et al.	2006	Asian	Hospital-based	108	238	48	60	104	134
Agudo et al.	2006	Caucasian	Population-based	242	927	120	122	434	498
Martinez et al.	2006	Caucasian	Population-based	87	329	54	33	180	149
Boccia et al.	2007	Caucasian	Hospital-based	105	256	48	59	119	135
Ruzzo et al.	2007	Caucasian	Population-based	79	112	44	35	51	61
Wideroff et al.	2007	Caucasian	Population-based	116	209	55	61	87	121
Tripathi et al.	2008	Caucasian	Population-based	76	100	45	31	61	39
Al-Moundhri et al.	2009	Caucasian	Population-based	107	107	65	42	75	32
Masoudi et al.	2009	Caucasian	Hospital-based	67	134	30	37	74	60
Malik et al.	2009	Caucasian	Hospital-based	108	195	44	64	116	79
Moy et al.	2009	Caucasian	Population-based	170	735	72	98	320	415
Zendehdel et al.	2009	Caucasian	Population-based	181	624	54	70	230	239
Palli et al.	2010	Caucasian	Population-based	296	546	206	90	271	275
Yadav et al.	2010	Asian	Hospital-based	133	270	84	49	150	120
Luo et al.	2010	Asian	Hospital-based	123	129	30	93	58	71
Nguyen et al.	2010	Asian	Hospital-based	59	109	16	43	34	75
Darazy et al.	2011	Caucasian	Hospital-based	13	70	7	6	58	12
García-González et al.	2012	Caucasian	Hospital-based	557	557	274	283	290	267
Malakar et al.	2012	Asian	Population-based	102	204	45	57	107	97
Jing et al.	2012	Asian	Hospital-based	410	410	170	240	203	207

### Quantitative synthesis

**Figure 2:**
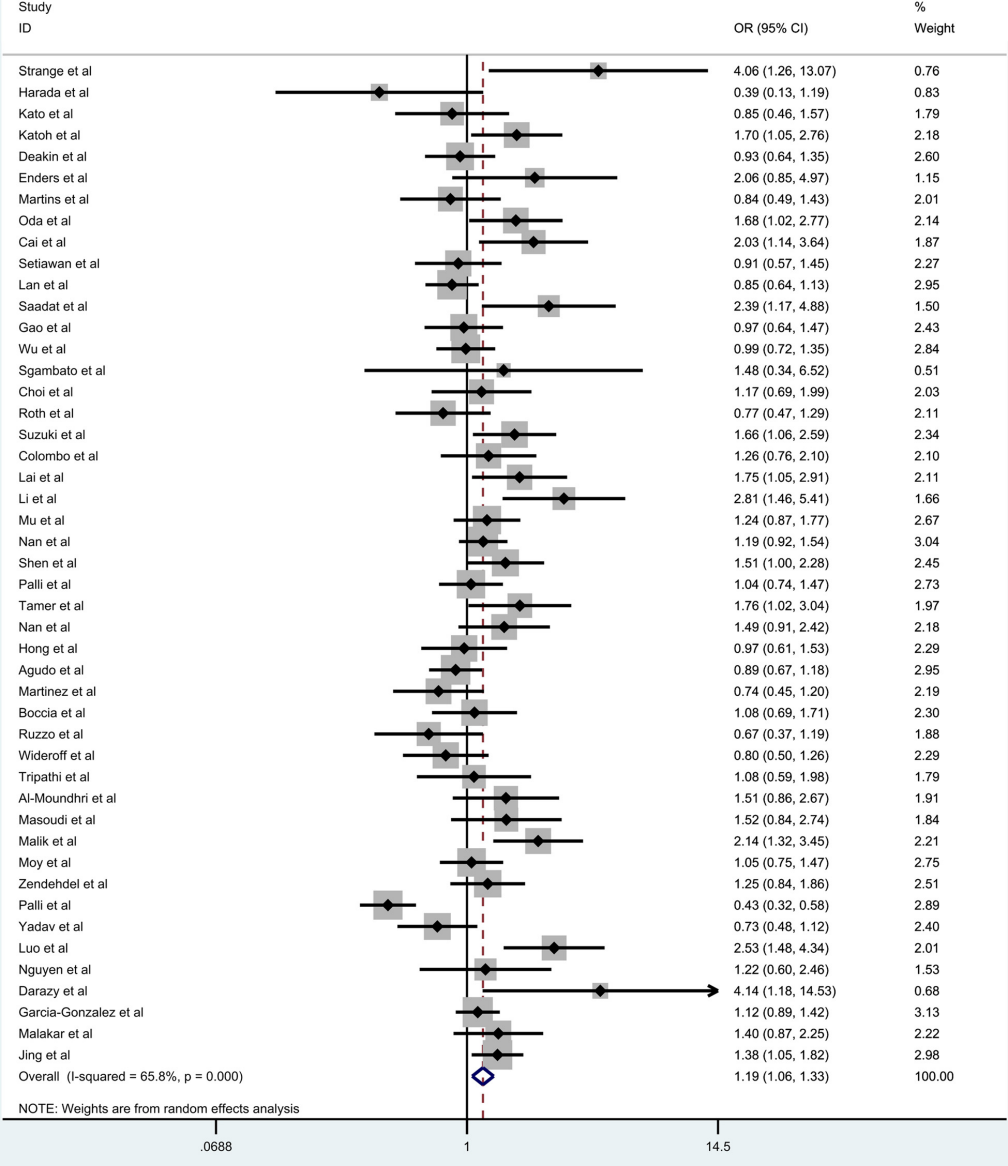
Meta-analysis of the association between GSTT1 null genotype and gastric cancer risk.

**Figure 3:**
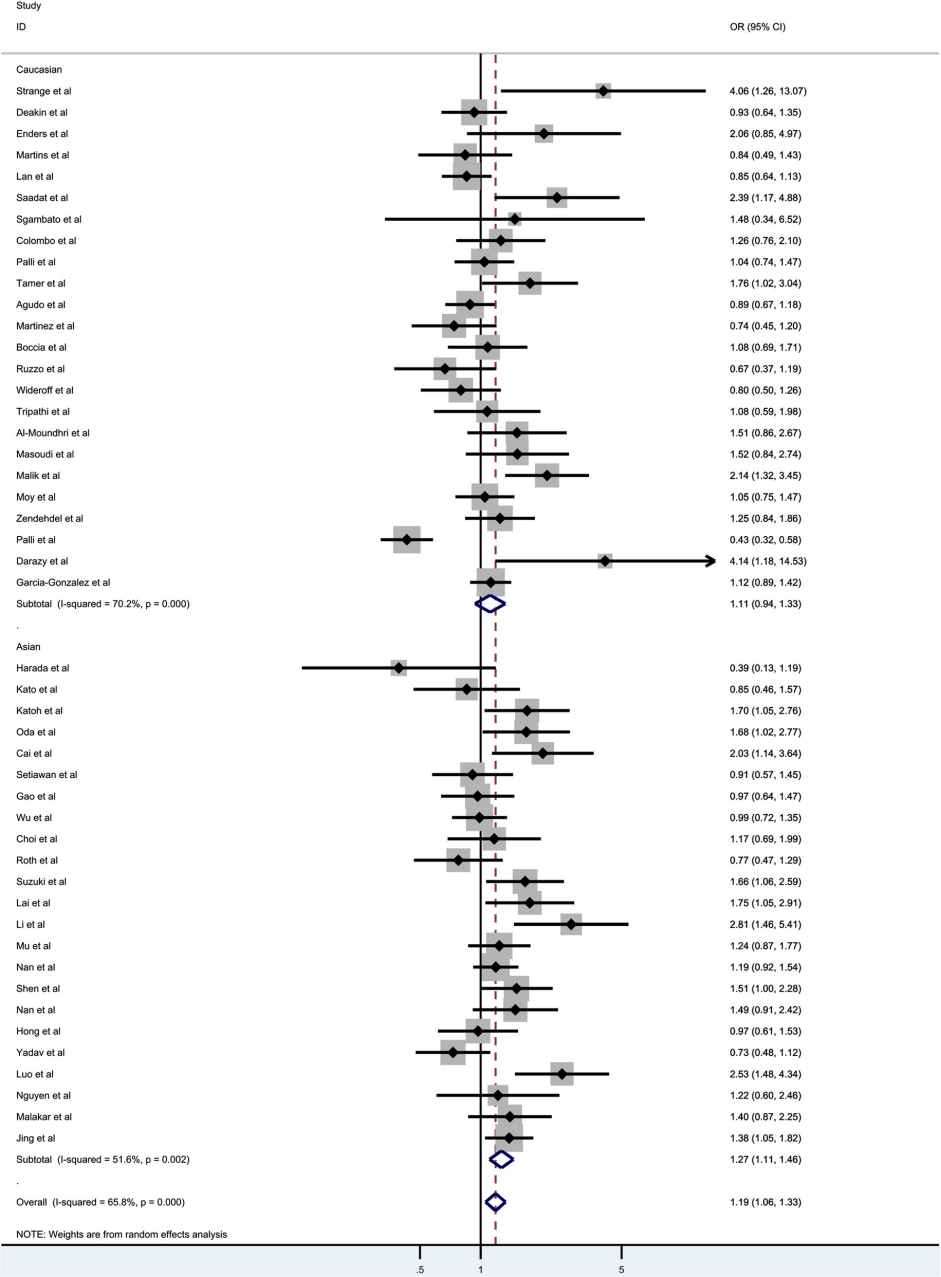
Subgroup analyses of the association between GSTT1 null genotype and gastric cancer risk by the ethnicity.

**Figure 4:**
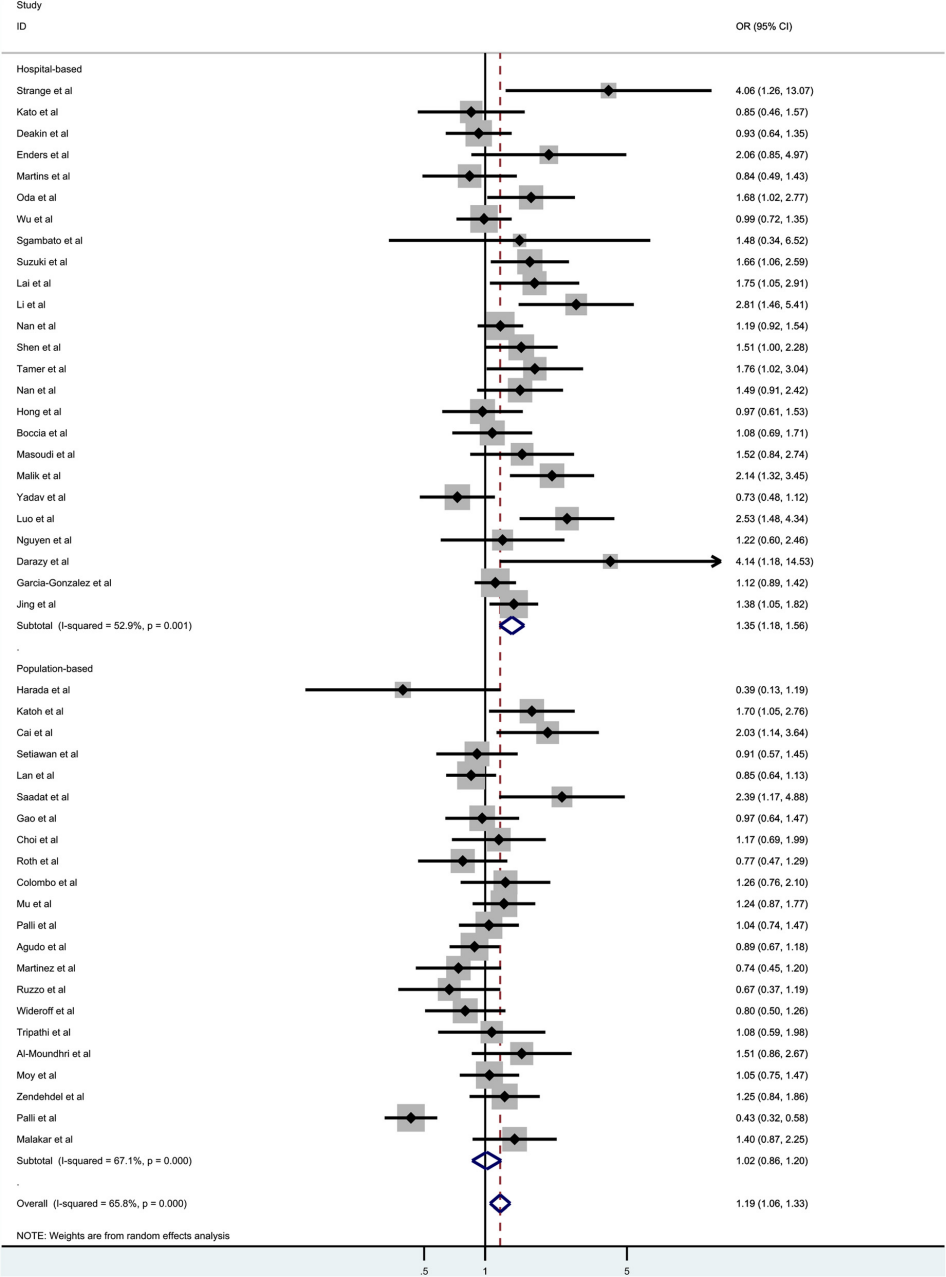
Subgroup analyses of the association between GSTT1 null genotype and gastric cancer risk according to the source of controls.

Overall, there was significant association between GC risk and the GSTM1 null genotypes when all the eligible studies were pooled into the meta-analysis (OR = 1.186, 95% CI = 1.057-1.329, P_heterogenetiy_ = 0.000, P = 0.004, Figure 2). Simultaneously, significant association was also found in Asians (OR = 1.269, 95% CI = 1.106-1.455, P_heterogenetiy_ = 0.002, P = 0.001, Figure 3). However, GSTM1 null genotype was not increased the risk of GC in Caucasians (OR = 1.115, 95% CI = 0.937-1.326, P_heterogenetiy_ = 0.000, P = 0.222, Figure 3). In the subgroup analysis stratified by sources of controls, significant association was detected in hospital-based studies (OR = 1.355, 95% CI = 1.179-1.557, P_heterogenetiy_ = 0.001, P = 0.000, Figure 4), while there was no significant association detected in population-based studies (OR = 1.017, 95% CI = 0.862-1.200, P_heterogenetiy_ = 0.000, P = 0.840, Figure 4).

### Sensitivity analysis

**Figure 5:**
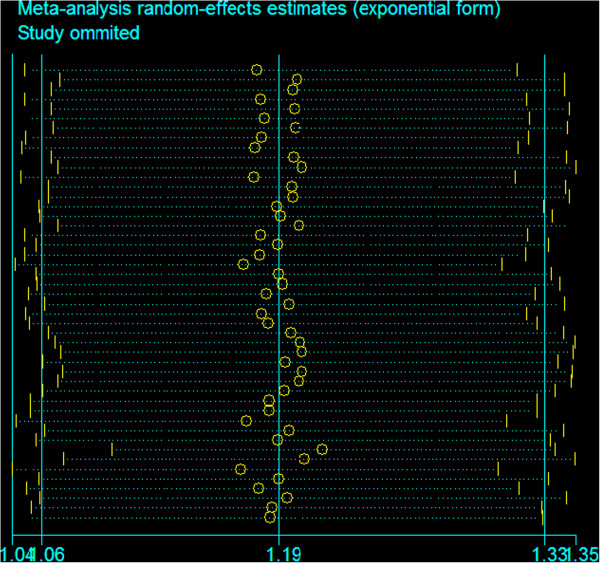
Sensitive analysis of the pooled ORs and 95% CI for the overall analysis, omitting each dataset in the meta-analysis.

In order to compare the sensitivity of this meta-analysis, we conducted a leave-one-out sensitivity analysis. A single study involved in this meta-analysis was evaluated each time to reflect the influence of the individual data set to pooled ORs. The results pattern was not impacted by single study (Figure 5).

### Publication bias

**Figure 6:**
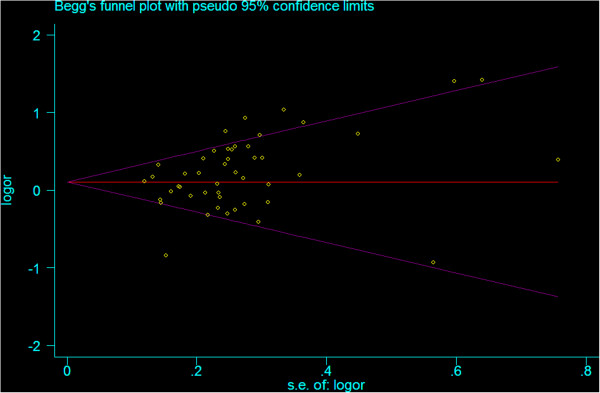
Begg’s test for detecting the potential publication bias.

Begg’s funnel plot and Egger’s test were used to assess the publication bias in this present work. The Funnel plots’ shape did not reveal obvious evidence of asymmetry (Figure 6), and the P value of Egger’s test was more than 0.05, providing statistical evidence for the funnel plots’ symmetry.

## Discussion

Gastric cancer is one of the most common malignancies in the world which accounts for 9.7% of total cancer deaths. Multiple factors have been proved contributed to the development of GC, including environmental, such as, Helicobacter pylori infection, Tobacco smoking and individual genetic polymorphism [63,64]. Since the first publication in 1991 by Strange et al. [17] reporting the association between the GSTT1 null genotype and the increased risk of GC, a large number of epidemiological studies concerning the link between GST gene polymorphisms and GC risk have been conducted. GSTM1 is generally considered as a protective enzyme because it detoxifies a number of toxic and carcinogenic substances such as nitrosamines and PAHs including BPDE [65].

As we all known, meta-analysis has great power to give a more credible results in one field than individual study through analyzing all the published research works with the same field [66,67]. Previous epidemiological studies have evaluated the association between the GSTM1 polymorphism and GC risk, but with inconclusive results. Therefore, it is necessary to perform this meta-analysis to identify the association between GSTM1 polymorphism and GC risk by combining the relevant studies published to date. Detection of gene genotype in all kinds of cancer not only in GC patient, which can be used for new therapeutic targets, will modify the current therapeutic approach. After pooling available data from all included studies, we found that there was significant association between this polymorphism and GC risk in over the world population. Our data are in line with those reported by Saadat et al. [68] and Boccia et al. [69] who observed a significantly increased risk of GC. This association can be explained by the reduced ability to detoxify the reactive intermediates that react with DNA because of the lack of GSTM1 enzyme activity [70].

It has been well known that cancer occurrence and mortality varied by ethnicity and geographic location. Piao et al. [71] suggested it was not associated with GC risk in different populations. In present work, significant association of GSTM1 polymorphism with GC risk was detected in Asian populations. However, no association was detected in Caucasians, which in line with previous meta-analysis conducted by Qiu et al. [72]. When stratified by source of controls, significant association between GSTM1 polymorphism and GC risk was observed among hospital-based studies. Many factors may contribute to this result, incompleteness of search, and include the potential false diagnoses (clinic, documentation, statistical methods). Furthermore, the use of typical control populations is vitally important, especially for the genetic association studies. The failure to reach a statistical significance in population-based studies implies that the selection of representative controls may reduce bias of the results.

Some limitations of this study should be acknowledged. Firstly, there was some heterogeneity in both the meta-analysis of total 48 studies and the subgroup analyses by ethnicity. The differences from the selection criteria of cases or controls, the adjusted confounding variables, and the ethnicity result in the heterogeneity. Secondly, most studies in the meta-analysis were retrospective design which could suffer more risk of bias owing to the methodological deficiency of retrospective studies. Those there was no obvious risk of publication bias in the present meta-analysis, the risks of other potential bias were unable to be excluded. Some misclassification bias was possible because most studies could not exclude latent gastric cancer cases in the control group. Therefore, more studies with prospective design and low risk of other bias are needed to provide a more precise estimate of the association between GSTM1 null genotype and GC risk. Finally, we could not address gene-gene and gene-environmental interactions in the association between GSTM1 null genotype and GC risk.

## Conclusion

In conclusion, the meta-analysis with all the eligible studies published up to now, provides a more precise evidence for the significant association between GSTM1 null genotype and increased risk of GC. In addition, more individual studies with well design are needed to further assess the possible gene-gene and gene-environmental interactions in the association between GSTM1 null genotype and GC risk.

## Competing interests

The authors declare that they have no competing interests.

## Authors’ contributions

XM, BL and YL conceived and designed the experiments. XM and BL analyzed the data. XM, BL and YL contributed reagents/materials/analysis tools. XM and YL wrote the paper. Yong Liu revised the paper. All authors read and approved the final manuscript.
